# The impact of social influence and perceived value on usage intention of county-level “Internet + Medical Health” services: a moderating analysis of region

**DOI:** 10.3389/fdgth.2026.1805619

**Published:** 2026-07-09

**Authors:** Lei Zhao, Lai Wei, Qingsong Zhang, Renfen Tian

**Affiliations:** 1 School of Public Health, Zunyi Medical University, Zunyi, Guizhou, China; 2 School of Management, Zunyi Medical University, Zunyi, Guizhou, China

**Keywords:** Internet + Medical health, perceived value, region, social influence, usage intention

## Abstract

**Objective:**

This study aims to explore the association of social influence and perceived value with usage intention of “Internet + Medical Health” services, and examine the model changes with region as a moderating variable, so as to explore key predictors of usage intention and examine regional moderation, with a view to informing targeted policy and practice recommendations.

**Methods:**

Based on the Technology Acceptance Model (TAM), an empirical study was conducted using questionnaire surveys. The data were analyzed and the structural equation model was constructed by AMOS software.

**Results:**

The results showed that patients' perceived value (*β* = 0.337, *P* < 0.05) and perceived usefulness (*β* = 0.303, *P* < 0.05) were positively associated with usage intention. Perceived value (*β* = 0.442, *P* < 0.05) and perceived ease of use (*β* = 0.382, *P* < 0.05) were positively associated with perceived usefulness. Perceived value was positively associated with perceived ease of use (*β* = 0.457*, P* < 0.05). Social influence was positively associated with perceived value (*β* = 0.307, *P* < 0.05). Perceived value partially mediated the positive association between social influence and usage intention. Perceived usefulness partially mediated the positive association between perceived ease of use and usage intention. Region moderated the association between social influence and perceived value.

**Conclusion:**

Social influence, perceived value, perceived ease of use and perceived usefulness are the key factors associated with patients' intention to use “Internet + Medical Health” services. Rural patients are more susceptible to social influence, which further adjusts their perceived value and changes their usage intention. To promote the in-depth integration of “Internet + Medical Health” services, it is necessary to coordinate technological ease of use, social communication and value cognition, focus on social guidance for rural patients, improve their health information literacy, and optimize the reduction of service costs of Internet-based medical care.

## 1 Introduction

As a product of the deep integration of advanced technology and traditional medical services, “Internet + Medical Health” has effectively broken geographical boundaries. It not only helps improve the efficiency of medical services and reduce patients' medical costs, but also further promotes the popularization, convenience, and personalization of medical services (1). In 2018, the General Office of the State Council issued the Opinions on Promoting the Development of “Internet + Medical Health”, making a comprehensive plan for the deep integration and development of the Internet and medical health (2). Leveraging technology as a lever, “Internet + Medical Health” drives the efficient integration and coordination of regional medical resources, provides tools and methods, and offers organizational frameworks and application scenarios for medical institutions. Together, they promote the upgrading of regional medical care from “fragmentation” to “systematization”, truly achieving the healthcare reform goal of “less running for patients, more running for data, and more inclusive resources”. County-level medical care undertakes the health demands of urban and rural residents, and the deep integration of “Internet + Medical Health” is gradually associated with changes in patients' medical ecology.

**Figure 1:**
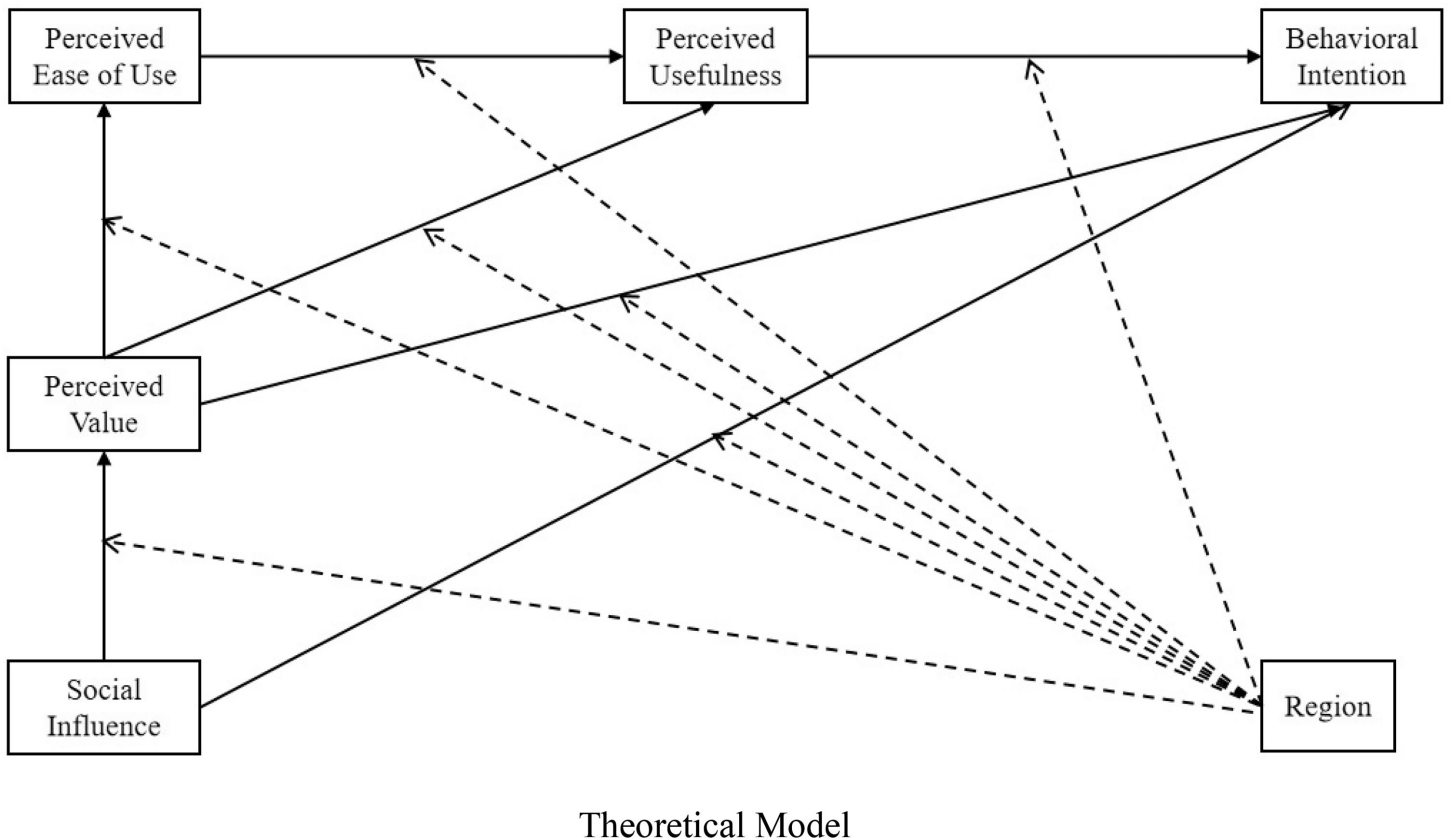
Theoretical model. PEOU, perceived ease of use; PU, perceived usefulness; BI, behavioral intention; PV, perceived value; SI, social influence.

The medical digital divide between urban and rural areas and regions is widespread globally. Studies in developed countries show that rural elderly groups and people with low education levels face problems such as insufficient digital literacy and low telemedicine utilization (3); in the promotion of digital primary medical care in developing countries, social influence, perceived value, and regional differences are also core factors associated with obstacles (4). Counties in China, especially western provinces such as Guizhou, represent a typical scenario for global medical digital divide governance. The conclusions of this study can provide references for the popularization of digital medical care at the primary level in developing countries. The World Health Organization (WHO) points out that the lack of digital health literacy policies and uneven regional development are widening the global medical digital divide, making vulnerable groups unable to benefit from digital medical care.

From the perspective of industry development, the coverage of Internet medical services in China continues to expand. As of June 2024, the number of Internet medical users in China reached 365 million, and telemedicine services have covered all counties nationwide (2, 5). Counties are the core carrier of China's primary medical services, and county-level medical institutions undertake a large number of diagnoses, treatments, and inpatient services for common and frequently occurring diseases among urban and rural residents, serving as a key link in the hierarchical diagnosis and treatment system (6). However, counties, especially rural areas, still face prominent problems such as insufficient digital literacy, low service cognition, low usage intention, and low actual utilization rate, and the urban-rural digital medical divide remains obvious (7, 8). Against this background, systematically exploring the factors associated with patients' usage intention of “Internet + Medical Health” services at the county level and clarifying the moderating effect of regional differences are of great practical significance for promoting service sinking and improving the accessibility of primary medical care.

## 2 Theoretical model and hypotheses

### 2.1 Definition of core research variables

Perceived usefulness refers to the degree to which patients believe that using county-level “Internet + Medical Health” services (Internet hospitals, online consultations, online drug purchases, report inquiries, chronic disease follow-up) can improve medical efficiency, save time costs, and enhance diagnosis, treatment, and health management effects. Perceived ease of use refers to the degree to which patients believe that the process of learning and operating the county-level “Internet + Medical Health” platform is simple, requires no complex skills, and is easy to master. Behavioral intention refers to patients' tendency to actively use, continuously use, and recommend others to use county-level “Internet + Medical Health” services in the future. The scale is derived from Davis's (9) classic TAM scale and adapted and revised locally in combination with the research context of Internet hospitals and online diagnosis and treatment.

Based on the Technology Acceptance Model (TAM) proposed by Davis (9), this study focuses on exploring the association of two main determinants, perceived usefulness and perceived ease of use, with behavioral intention. The TAM model can be used to study the relationships between variables in any acceptable technology context. Relevant domestic studies based on the TAM model show that users' perceived ease of use and perceived usefulness are positively associated with their behavioral intention (10, 11). Mo et al. empirically showed that the ease of use of online consultation platforms is positively associated with behavioral intention through perceived usefulness (12). Therefore, the following hypotheses are proposed:

- H1: Perceived ease of use is positively associated with users' perceived usefulness (PEOU → PU).
- H2: Perceived usefulness is positively associated with users’ usage intention (PU → BI).
- H3: Perceived ease of use is indirectly positively associated with users' usage intention through perceived usefulness (PEOU → PU → BI).

Perceived value is the overall evaluation of the utility of a product or service by customers after weighing the benefits they can perceive against the costs they pay when obtaining the product or service. With the deepening and expansion of research, perceived value is a key factor associated with users' decision-making process of whether to purchase or use a product (11), which has been confirmed by many studies at home and abroad (12, 13). The scale is compiled based on the customer perceived value theory and combined with Internet medical value perception research. Tai et al. divided perceived value into positive guidance, internal imitation, and construction of value, and concluded that all three parts are positively associated with perceived ease of use and perceived usefulness (14). Wang et al. found that perceived value is positively associated with Internet users' behavioral intention based on the TAM model (15). The above research fields are similar to the “Internet + Medical Health” service field of this study. Therefore, the following hypotheses are proposed:

- H4: Perceived value is positively associated with perceived ease of use (PV → PEOU).
- H5: Perceived value is positively associated with perceived usefulness (PV → PU).
- H6: Perceived value is positively associated with usage intention (PV → BI).

Social influence is a very common social psychological phenomenon, referring to the process in which an individual's behavior and attitude change toward the dominant direction of society due to social pressure. The scale is revised based on Kelman's social influence theory and referring to Internet medical and primary medical technology adoption research. Kelman proposed three processes of social influence: compliance, identification, and internalization (16), indicating that people's behavioral intention and acceptance of unknown things are often associated with social influence. Many studies have empirically verified the association of social influence with users' behavioral intention in different information technology fields such as e-government, mobile payment, and open educational resources (17–19). For example, social influence of e-reading platform users on clients is associated with behavioral intention and perceived value, and perceived value partially mediates the association between social influence and behavioral intention (20). The above research fields are similar to the “Internet + Medical Health” service field of this study. Therefore, the following hypotheses are proposed:

- H7: Social influence is positively associated with usage intention (SI → BI).
- H8: Social influence is positively associated with perceived value (SI → PV).
- H9: Social influence is indirectly associated with usage intention through perceived value (SI → PV → BI).

Region usually refers to a certain geographical space, a complex formed by the interaction of natural and human factors. People in different regions have different associations with social influence. Studies by Ma et al. show that there are regional differences in the continuous behavioral intention of platforms, and rural users have higher behavioral intention than urban users (21). Perceived value is more significantly positively affected by region (22). This paper holds that there are many scenarios of “Internet + Medical Health” services, and regional differences are associated with patients using such services in various aspects. Therefore, the following hypothesis is proposed:

- H10: Region significantly moderates the core associations of the theoretical model.

### 2.2 Theoretical limitations

Most TAM-based studies focus on direct associations of perceived usefulness/ease of use and rarely integrate social influence, perceived value, and regional moderation simultaneously in county-level healthcare contexts (23, 24); research on the regulatory mechanism of urban-rural differences is insufficient, especially lacking empirical evidence that rural patients are positively related to social influence (25, 26). From the perspective of county-level patients, this paper describes the usage intention of “Internet + Medical Health” services, constructs a model, and predicts patients' usage intention of such services from the perspectives of social influence and perceived value, as well as the moderating effect of region, so as to provide a decision-making basis for promoting the deep integration of “Internet + Medical Health”.

While prior technology acceptance studies (e.g., UTAUT/UTAUT2) include social influence and facilitating conditions, they are rarely applied to county-level primary healthcare in China. This study retains TAM as the core framework because it provides a parsimonious foundation for healthcare technology adoption, and extends it by integrating perceived value, social influence, and regional moderation—a three-way integration not yet systematically examined in rural–urban digital health disparities in China.

## 3 Research objects and methods

### 3.1 Research objects and general information

The researcher designed the questionnaire by consulting literature, and conducted a pre-survey after expert review and demonstration. The pre-survey was conducted in Wudang District People's Hospital and Traditional Chinese Medicine Hospital of Guiyang City, Guizhou Province, with a total of 62 questionnaires distributed. The questionnaire design was improved through pre-survey and data analysis.

The formal survey adopted stratified random sampling. From September to November 2024, paper questionnaires on the behavioral intention of “Internet + Medical Health” services were distributed to patients in People's Hospitals and Traditional Chinese Medicine Hospitals of Xishui County (Zunyi City), Wudang District (Guiyang City), and Sinan County (Tongren City), Guizhou Province. Paper questionnaires were randomly distributed offline, and the survey was conducted anonymously, with one-to-one distribution and on-site collection. The sample was recruited from three counties in Guizhou Province, limiting geographic generalizability. Participants were hospital outpatients/inpatients, who may exhibit higher health engagement than the general population.

A total of 488 questionnaires were collected. After eliminating invalid questionnaires with logical errors and missing information, 460 valid questionnaires were finally obtained, with an effective rate of 94.26%. According to statistical analysis requirements, the sample size meets the analysis needs of this study. The respondents were mainly male (59.78%), geographically concentrated in rural areas (56.3%), with agricultural workers accounting for the highest proportion (54.57%), and married groups accounting for an absolute majority (85.65%). The survey subjects were dominated by low-income groups, 60.22% had a personal monthly income of less than 1,500 yuan, 51.96% had primary school education or below, and 82.39% had junior high school education or below. 28.48% of the respondents suffered from chronic diseases, of which 33.59% and 53.44% rated their conditions as “relatively severe” and “moderate” respectively. The type of medical treatment was mainly inpatient (65.22%), and internal medicine was the most common department (50.80%). Nonresponse bias was assessed by comparing early (first 25%) and late (last 25%) respondents on key demographic variables and main constructs. No significant differences were observed (*P* > 0.05), suggesting minimal nonresponse bias.

### 3.2 Variable measurement

The questionnaire in this study includes three parts. The first part is basic information and general conditions; the second part is the current medical treatment situation; the third part is the scale of behavioral intention of “Internet + Medical Health” services (including nine dimensions such as usefulness, ease of use, value selection, and social influence). All scales were scored using a 5-point Likert scale, with values assigned from 1 to 5 in order from “completely inconsistent” to “completely consistent”.

### 3.3 Data analysis software and tools

This study adopted multi-software collaboration to complete data processing and analysis, with clear purposes and versions of each software: (1) SPSS 26.0: Completed descriptive statistics, reliability test (Cronbach's *α*), validity test (KMO, Bartlett's sphericity test), Pearson correlation analysis, and common method bias test; (2) AMOS 27.0: Constructed structural equation model (SEM), confirmatory factor analysis (CFA), path coefficient test, mediating effect analysis, and multi-group moderating effect analysis; (3) Process 4.0 plug-in (integrated in SPSS 26.0): Assisted in verifying the robustness of mediating effects and cross-validating with AMOS results.

Common method bias (CMB) was assessed using Harman's single-factor test. Exploratory factor analysis indicated that the first factor explained 38.7% of the total variance, below the 50% threshold, suggesting no severe CMB. A common latent factor was also included in the SEM, and the model fit change was minimal (Δ*χ*^2^ = 12.4, Δdf = 5, *P* > 0.05), confirming that CMB was not a major concern in this study.

## 4 Research results

### 4.1 Reliability test

The internal consistency of each dimension was verified by Cronbach's alpha reliability test. The Cronbach's alpha coefficient of the total scale was 0.931, and the Cronbach's alpha coefficient of each dimension was above 0.7, indicating good reliability (Table 1).

**Table 1:** Confirmatory factor analysis and reliability.

Latent variable	Observed variable	Unstandardized loading estimate	S.E.	C.R.	*P*	Standardized loading estimate	Cronbach's *α*	CR	AVE
PU	PU1	1	–	–	–	0.84	0.736	0.789	0.568
PU	PU2	1.863	0.096	19.455	0	0.867			
PU	PU3	0.691	0.065	10.568	0	0.497			
PEOU	PEOU1	1	–	–	–	0.933	0.936	0.9	0.964
PEOU	PEOU2	1.08	0.025	43.615	0	0.967			
PEOU	PEOU3	1.117	0.028	40.238	0	0.946			
PV	PV1	1	–	–	–	0.916	0.96	0.893	0.961
PV	PV2	1.124	0.029	39.159	0	0.96			
PV	PV3	1.159	0.03	38.878	0	0.958			
SI	SI1	1	–	–	–	0.881	0.919	0.752	0.923
SI	SI2	1.07	0.033	32.913	0	0.97			
SI	SI3	0.962	0.037	26.231	0	0.862			
SI	SI4	0.912	0.046	19.756	0	0.738			
BI	BI1	1	–	–	–	0.97	0.957	0.912	0.969
BI	BI2	1.06	0.017	61.164	0	0.98			
BI	BI3	1.274	0.031	41.157	0	0.914			

### 4.2 Validity test

The KMO value of the scale for the influencing factors of usage intention of “Internet + Medical Health” services was 0.861, and the *χ*^2^ value of Bartlett's test of sphericity was 8,049.237 (*P* < 0.001), indicating the existence of differences among variables and the suitability for factor analysis.

The maximum variance method was used to extract common factors with eigenvalues greater than 1. A total of 5 common factors were extracted, and the cumulative variance contribution rate was 87.535%, indicating that the construct validity of the questionnaire passed the test.

Confirmatory factor analysis was conducted according to the proposed hypotheses. The standardized factor loadings of all measurement indicators on latent variables were greater than 0.5, which were significant. The critical ratio (C.R.) was greater than 3.29, passing the significance test at the 0.001 level. Each variable had a strong explanatory power for the model, and the model fit well. The *CR* values of all variables were greater than 0.7, and the *AVE* values were greater than 0.5, meeting the standards of convergent validity (Table 1).

### 4.3 Correlation analysis

In terms of discriminant validity, Pearson's rank correlation coefficient was used in this study to measure the correlation between variables. If the correlation coefficient between variables is less than the square root of the corresponding *AVE* value of the variables, the questionnaire has good discriminant validity. The test results showed that the questionnaire had good discriminant validity (Table 2).

**Table 2:** Square root of *AVE* of latent variables and correlation coefficients between variables.

Variable	PU	PEOU	PV	SI	BI
PU	0.754				
PEOU	0.514	0.949			
PV	0.553	0.440	0.945		
SI	0.002	0.145	0.229	0.867	
BI	0.472	0.473	0.527	0.254	0.955

PU, perceived usefulness; PEOU, perceived ease of use; PV, perceived value; SI, social influence; BI, behavioral intention.

High factor loadings (0.93–0.98) and *AVE* values (0.94–0.96) reflect high item homogeneity and strong unidimensionality of each construct, which is acceptable in well-defined, narrow constructs in health technology research. No redundant items were removed because all items were theoretically meaningful and retained in the original scale design.

Discriminant validity was further confirmed using the Heterotrait–Monotrait Ratio (HTMT). All HTMT values were <0.85, supporting adequate discriminant validity (Table 3).

**Table 3:** Heterotrait–Monotrait ratio (HTMT) for discriminant validity.

Variable	PU	PEOU	PV	SI	BI
PU	–				
PEOU	0.480	–			
PV	0.510	0.420	–		
SI	0.180	0.130	0.210	–	
BI	0.450	0.440	0.500	0.230	–

### 4.4 Model fitting and hypothesis testing

The model was established and modified using AMOS 27.0. The path coefficients and correlations showed that all *P* < 0.05, and the hypotheses were initially established. The model fit was tested as follows: *χ*^2^*/df* = 4.646 < 5, *CFI* = 0.957, *TLI* = 0.946, *SRMR* = 0.086, *RMSEA* = 0.089, *NFI* = 0.946, *NNFI* = 0.946. Model fit indices were acceptable: *χ*^2^*/*df = 4.646, *CFI* = 0.957, *TLI* = 0.946, *RMSEA* = 0.089 (marginal acceptable), *SRMR* = 0.086 (borderline acceptable) (27, 28).

### 4.5 Path coefficient analysis of structural equation model

**Figure 2:**
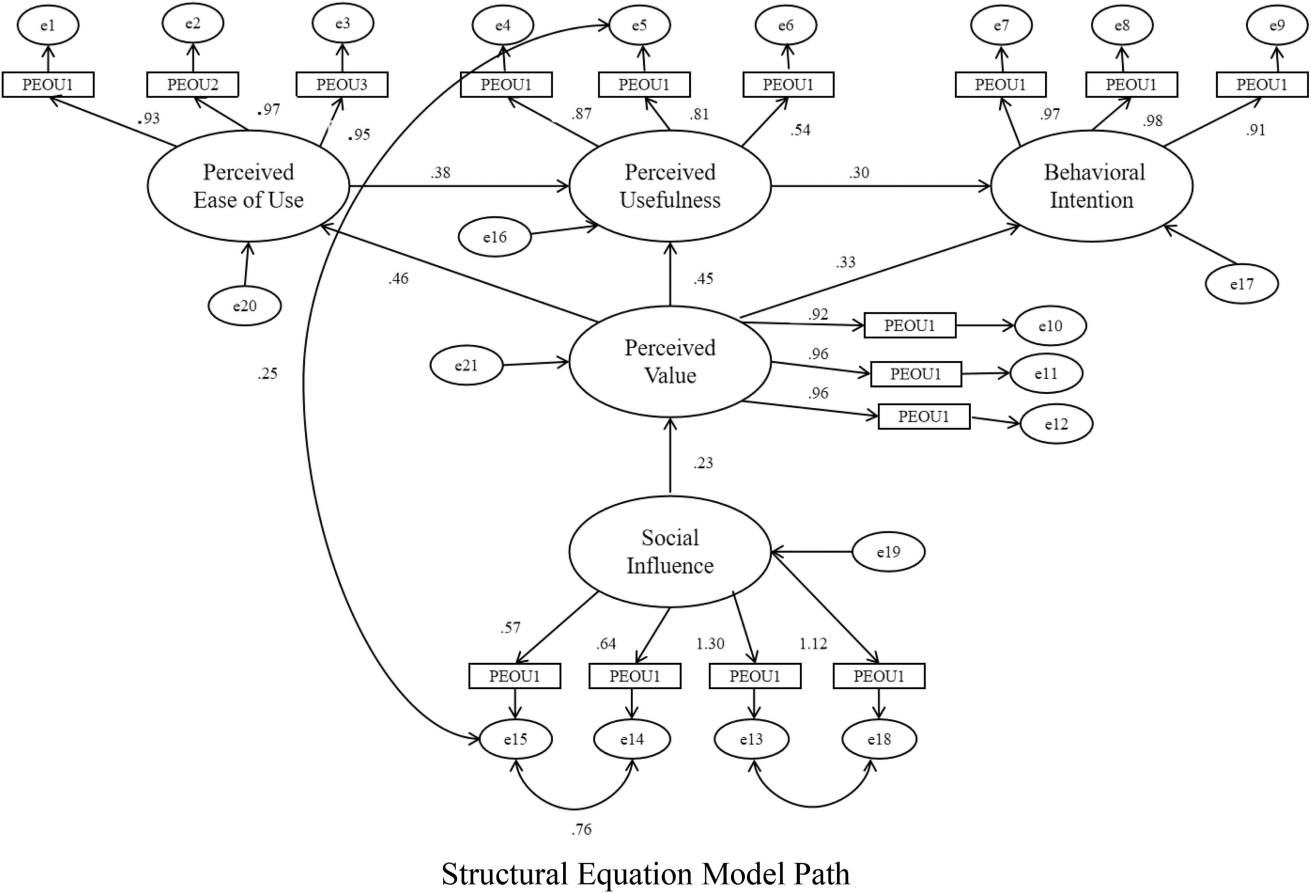
Structural equation model path. PEOU, Perceived ease of use; PU, perceived usefulness; BI, behavioral intention; PV, perceived value; SI, social influence.

According to Table 4 and Figure 2, perceived value was significantly positively associated with perceived usefulness, with a standardized coefficient of 0.442 (*P* < 0.05); perceived value was significantly positively associated with perceived ease of use, with a standardized coefficient of 0.457 (*P* < 0.05); perceived value was significantly positively associated with behavioral intention, with a standardized coefficient of 0.337 (*P* < 0.05); perceived usefulness was significantly positively associated with behavioral intention, with a standardized coefficient of 0.303 (*P* < 0.05); perceived ease of use was significantly positively associated with perceived usefulness, with a standardized coefficient of 0.382 (*P* < 0.05); social influence was significantly positively associated with perceived value, with a standardized coefficient of 0.307 (*P* < 0.05); social influence is mostly indirectly associated with behavioral intention, with a standardized coefficient of 0.060 (*P* > 0.05), and the modification index showed that this path had limited improvement on model fitting. Combined with empirical conclusions in the field of Internet medical care, social influence mostly acts indirectly on behavioral intention (15, 20). This path was deleted and the model was re-estimated. The direct path from social influence to usage intention (H7) was non-significant (*β* = 0.060, *P* = 0.061). Given the theoretical argument that social influence operates primarily through perceived value in healthcare contexts, and to improve model parsimony, this path was removed. The final model retained all theoretically justified paths. The modified model had good fit indicators and was acceptable. Therefore, all hypotheses were supported except H7.

**Table 4:** Path coefficient estimation of structural equation model.

*X*	→	*Y*	Unstandardized regression coefficient	Standardized regression coefficient	S.E.	*Z* (C.R.)	*P*
Perceived value	→	Perceived Usefulness	0.349	0.442	0.037	9.463	<0.05
Perceived value	→	Perceived Ease of Use	0.458	0.457	0.045	10.197	<0.05
Perceived value	→	Usage Intention	0.357	0.337	0.058	6.189	<0.05
Perceived usefulness	→	Usage Intention	0.406	0.303	0.078	5.238	<0.05
Perceived ease of use	→	Perceived Usefulness	0.300	0.382	0.036	8.296	<0.05
Social influence	→	Perceived Value	0.338	0.307	0.051	6.618	<0.05
Social influence	→	Usage Intention	0.040	0.060	0.023	1.873	0.061

The model explained substantial variance in key constructs: *R*^2^ (BI) = 0.42, *R*^2^ (PU) = 0.38, *R*^2^ (PV) = 0.29, *R*^2^ (PEOU) = 0.21.

### 4.6 Mediating effect

Mediation analysis was conducted with 5,000 bootstrap samples. Partial mediation was confirmed: the direct effect remained significant after including mediators, and indirect effects were statistically significant (95% CI excluded zero). The associations of perceived ease of use and perceived value of “Internet + Medical Health” services with usage intention were partially mediated by perceived usefulness; the associations of social influence and perceived value of such services with usage intention were partially mediated by perceived value and perceived ease of use respectively (Table 5).

**Table 5:** Mediating effect analysis of the model.

Item	Effect value	95% CI		*P*
		Lower limit	Upper limit	
Social influence → perceived value → usage intention	0.196	0.164	0.283	<0.05
Perceived value → perceived usefulness → usage intention	0.155	0.088	0.203	<0.05
Perceived value → perceived ease of use → usage intention	0.142	0.089	0.176	<0.05
Perceived ease of use → perceived usefulness → usage intention	0.177	0.11	0.213	<0.05

### 4.7 Moderating effect analysis of the model

Multi-group analysis was conducted using AMOS 27.0 to judge the significance of “region” on the model. According to the method proposed by Wen Zhonglin et al. (29), the sample was divided into urban and rural groups, and the regression coefficients of the two groups were all restricted to be equal to obtain the *χ*^2^ and degrees of freedom of the model. The structural equation regression coefficients of the two groups were restricted to be equal to obtain a *χ*^2^ value and corresponding degrees of freedom. Then the restriction was removed, the model was re-estimated, and another *χ*^2^ value and corresponding degrees of freedom were obtained. A new *χ*^2^ was obtained by subtracting the latter *χ*^2^ from the former, and its degrees of freedom were the difference between the degrees of freedom of the two models. If the *χ*^2^ test result was statistically significant, the moderating effect was significant.

It can be seen from the unrestricted model and factor loading model that *P* < 0.05, indicating that urban and rural areas have a moderating effect on the model (Table 6). According to the judgment criteria given by Cohen et al. (30), 1.96 was taken as the significant critical ratio, and the underlined data was 3.173 > 1.96 (Table 7), so it had a moderating effect on path AW4 (SI → PV). For the path SI → PV, the standardized coefficient was significantly higher in rural groups (*β* = 0.07, *P* < 0.05) than in urban groups (*β* < 0.001, *P* > 0.05) (Table 8), indicating that rural patients were more susceptible to social influence (friends, media, policies, etc.) to make value judgments on “Internet + Medical Health” services.

**Table 6:** Significance of restrictions.

Model	DF	CMIN	*P*
Unconstrained model vs. factor loading model	11	30.167	0.001
Factor loading model vs. path coefficient (regression coefficient) congruent model	5	3.934	0.559
Path coefficient congruent model vs. structural covariance model	5	15.716	0.008
Structural covariance model vs. structural residual model	19	59.43	***

*** *P* < 0.001.

**Table 7:** Critical ratio matrix of path coefficients.

Label	AW1	AW2	AW3	AW4	AW5	AW6	AW7	AW8
BW1	−1.187							
BW2	−1.199	1.264						
BW3	−1.187	0.809	0.662					
BW4	−7.711	−4.812	−2.755	−3.173				
BW5	−2.76	−0.232	−0.12	−0.065	−0.783			
BW6	−1.33	0.384	0.346	0.426	−0.056	−0.825		
BW7	9.888	13.55	8.602	10.062	10.556	4.257	1.421	
BW8	9.578	13.096	8.466	9.861	10.297	4.216	1.315	−0.304

**Table 8:** Moderating effect of different regions on paths.

Path	Rural		Urban	
	*P*	Label	*P*	Label
Perceived value ← social influence	0.07	BW4	***	AW4
Perceived ease of use ← perceived value	***	BW1	***	AW1
Perceived usefulness ← perceived value	***	BW2	***	AW2
Perceived usefulness ← perceived ease of use	***	BW5	***	AW5
Usage intention ← perceived value	***	BW3	0.002	AW3
Usage intention ← perceived usefulness	***	BW6	0.001	AW6

*** *P* < 0.001.

Measurement invariance testing (configural, metric, scalar) was not performed due to sample size constraints. Group comparisons should be interpreted with caution. Effect size (Cohen's *f*^2^, Δ*R*^2^) for moderation was not calculated due to software limitations; future studies should report these indices.

## 5 Discussion

### 5.1 Perceived value positively affects perceived ease of use, perceived usefulness and usage intention

According to the model path diagram, the path coefficients of perceived value with perceived ease of use, perceived usefulness, and behavioral intention were 0.457, 0.442, and 0.337 respectively, indicating that perceived value was positively associated with perceived ease of use, perceived usefulness and behavioral intention. Mediation analysis showed that the effect of perceived value's association with behavioral intention was partially mediated by perceived ease of use and perceived usefulness. This is consistent with the finding that users' perceived value in the service industry is significantly positively associated with their behavioral intention, and behavioral intention increases with the increase of users' perceived value (31, 32). Patients' perceived value of “Internet + Medical Health” services mainly comes from two aspects: one is the matching degree between the price and service of such services and their own acceptance; the other is the advantage of the price of such services compared with offline services. Therefore, improving patients' behavioral intention of “Internet + Medical Health” services should start from three aspects: perceived value, perceived ease of use, and perceived usefulness.

### 5.2 Perceived ease of use is positively associated with perceived usefulness

According to the model path diagram, the path coefficient of perceived ease of use and perceived usefulness was 0.382, indicating the impact of perceived ease of use on perceived usefulness. This is consistent with the finding that users' perceived ease of use is positively associated with perceived usefulness in Internet services, and perceived usefulness increases with the increase of perceived ease of use (33, 34). Patients' perceived ease of use of “Internet + Medical Health” services mainly comes from two parts: one is that such services are easy to accept and use; the other is that patients' own conditions prevent them from using such services. Therefore, improving patients' perceived usefulness of “Internet + Medical Health” services can be achieved by enhancing ease of use.

### 5.3 Social influence is positively associated with perceived value

According to the model path diagram, the path coefficient of social influence and perceived value was 0.307, indicating that social influence positively affected perceived value. Mediation analysis showed that social influence's association with behavioral intention was partially mediated by perceived value. Patients' social influence on “Internet + Medical Health” services mainly comes from four parts: opinions of relatives and friends, recommendations from medical staff, national policy support, and media advertising. This indicates that social identity and external environment are important driving forces for users to accept new services. Social influence is first associated with stronger users' value perception (believing that services have high cost performance and acceptable costs), and this value recognition is further associated with actual behavioral intention. This shows that relying solely on external publicity or policy guidance may not be enough, and it is also necessary to ensure that users truly recognize the value attributes of services.

### 5.4 The moderating role of region

According to the model moderating effect analysis, grouping by region found that the path coefficient (0.07) of rural region for SI → PV was significantly larger than that of urban region (<0.001), indicating that regional factors played a significant moderating role in the positive association between social influence and perceived value, and regional difference was a key boundary condition associated with the relationship between social role and value judgment. These patterns may be related to lower digital literacy, traditional healthcare reliance, and stronger social network dependence in rural areas, though these factors were not directly measured in this study. Compared with rural patients, urban patients have lower or fewer of the above reasons, and they may pay more attention to non-price factors such as service quality and privacy protection.

### 5.5 Mechanism explanation for the non-significant direct effect of social influence on behavioral intention

The results of this study show that the direct association between social influence and behavioral intention did not reach a significant level (hypothesis H7 is not supported). This result is not caused by model setting bias or measurement errors, but is highly consistent with the scenario characteristics of county-level Internet medical care and patients' behavioral laws, which can be analyzed from three dimensions:

First, patients in county-level and rural areas have long been accustomed to offline medical treatment models, and still have obvious concerns about the safety of online diagnosis and treatment services and the convenience of medical insurance reimbursement. External publicity and promotion have weak direct associations with changes in their inherent medical treatment habits. Only after patients fully recognize the actual value of online services can they form behavioral intention (7). It can be seen that social influence can only have a certain association with patients' cognitive judgment, but cannot directly promote their formation of actual behavioral tendency.

Second, the current publicity work on county-level Internet medical care mostly focuses on macro policy interpretation and technical advantage elaboration, which is out of line with the core demands of county-level patients such as low cost, easy operation, and reimbursable expenses, resulting in the difficulty of social influence being effectively transformed into patients' behavioral intention (35).

Third, medical services are highly professional and have high health risks, which are essentially different from ordinary information technology services. Patients will not choose to use online medical services solely based on others' recommendations, but will form corresponding behavioral intentions only after a comprehensive evaluation of service value and practicality.

## 6 Conclusions

### 6.1 Suggestions based on perceived value findings

Clearly mark service fees to avoid hidden charges, and launch low-price packages for high-frequency needs (such as chronic disease management); promote operation guidance videos and carry out offline training in communities, focusing on covering the elderly and rural groups; access AI-assisted diagnosis, open the whole process of “consultation-prescription-dispensing-payment”, and realize one-stop services. Improve patients' behavioral intention of services through the coordination of price transparency, simplified processes, and specialized functions.

### 6.2 Suggestions based on perceived ease of use findings

Simplify operation processes, develop one-stop registration and intelligent guidance functions, achieve multi-terminal compatibility, and lower user thresholds; popularize service advantages, organize community activities, carry out health lectures, and guide medical staff to take the initiative to recommend; develop customized functions, improve chronic disease follow-up reminders and health data synchronization modules, and adapt multi-language/accessible modes; optimize service processes, improve technological usability, strengthen user support systems, and lower patients' operation thresholds. Multiple measures are taken to help patients more intuitively feel the convenience of “Internet + Medical Health” services (such as reducing queuing and remote diagnosis and treatment), thereby recognizing their actual value (perceived usefulness) and promoting the popularization of such services and improving the accessibility of medical resources and patients' behavioral intention.

### 6.3 Suggestions based on social influence research results

Strengthen social influence through multi-channel publicity (such as policy advocacy, word-of-mouth from relatives and friends, media publicity), and highlight the price advantage and value matching of services to enhance users' value selection tendency; continuously optimize the cost performance and user experience of online services to ensure that users' perceived value is consistent with the information transmitted by social influence, thereby improving conversion rate; simultaneously strengthen security measures in promotion to reduce users' concerns about privacy and functional perfection, so as to reduce the risk of social influence being offset by negative factors.

### 6.4 Suggestions targeted at regional moderation characteristics

Combine the language habits of rural patients, expand publicity channels (such as dialect promotional videos, village-level broadcasts) to explain policy dividends; highlight the price advantage of online services (such as reducing transportation costs), and enhance value perception through case comparisons between patients' online medical treatment and traditional medical treatment; promote information literacy training action plans to focus on improving residents' digital capabilities, especially vulnerable groups; strengthen the publicity of doctor qualifications, optimize online consultation processes, and reduce dependence on “social influence”; improve the promotion of services by primary medical staff and community opinion leaders (such as village doctors), use authority effect to enhance trust in online medical treatment, and take multiple measures to further improve rural patients' behavioral intention of “Internet + Medical Health” services.

### 6.5 Core research conclusion summary

Social influence, perceived value, perceived ease of use and perceived usefulness are the key factors associated with patients' intention to use “Internet + Medical Health” services. Rural patients are more susceptible to social influence, which further adjusts their perceived value and changes their usage intention. To promote the in-depth integration of “Internet + Medical Health” services, it is necessary to coordinate technological ease of use, social communication and value cognition, focus on social guidance for rural patients, improve their health information literacy, and optimize the reduction of service costs of Internet-based medical care.

## 7 Implications

### 7.1 Theoretical implications

1. Expand the application boundary of the TAM model in county-level Internet medical scenarios. This study integrates perceived value, social influence, and regional moderating variables into the classic TAM model, and empirically verifies the explanatory power of the integrated model in primary medical scenarios, filling the theoretical gap that existing studies mostly focus on urban scenarios and ignore differences in county-level and rural areas. It provides new empirical evidence for the application of the TAM model in the field of medical digitalization and among vulnerable groups at the primary level.
2. Clarify the internal mechanism of social influence transmission to behavioral intention. The results show that social influence is not directly associated with behavioral intention, but is indirectly associated through the partial mediating effect of perceived value. This finding clarifies the key mediating role of perceived value in the transformation from external social factors to individual behavioral intention, and further improves the core theoretical logic chain of “external environment—individual cognition—behavioral intention”.
3. Reveal the boundary moderating effect of regional factors on the action path of social influence. This study empirically verifies for the first time at the county level that urban-rural regional differences significantly moderate the effect of social influence on perceived value, and rural patients are more easily driven by social influence. This conclusion advances technology adoption research from a single individual cognitive level to an interaction level between individuals and the environment, enriching the situational moderation theory in the field of medical digital adoption.
4. Deepen the scenario adaptability research of the core chain mediation of the TAM model. The study verifies the existence of the chain mediation relationship of “perceived ease of use → perceived usefulness → behavioral intention”, and finds that perceived ease of use has a more prominent driving effect on perceived usefulness among county-level patients, providing new empirical support for the application of the TAM model in groups with relatively low digital literacy.

### 7.2 International implications

The empirical findings of this study based on China's county-level scenarios echo relevant research in the global digital health field. The report released by the World Health Organization (WHO, 2023) points out that a large number of rural and vulnerable groups worldwide cannot conveniently access telemedicine services due to factors such as low digital literacy, social support conditions, and regional development differences. The core dilemmas are consistent with the conclusions of this study, namely insufficient perceived value, low efficiency of social influence transmission, and obvious urban-rural digital literacy gaps. In low- and middle-income countries such as India, Thailand, and Tanzania, rural areas also face the problems of “low technology promotion difficulty and low user acceptance”. Low digital literacy, dependence on social networks, and regional development differences all significantly affect local residents' adoption intention of digital medical services. This study confirms that perceived value and perceived ease of use are cross-scenario general key influencing factors, and the regional moderating effect has global reference significance, providing empirical references from China's counties for countries around the world to narrow the medical digital divide and promote digital medical services to cover rural and vulnerable groups.

## 8 Limitations and prospects

Limitations: (1) The sample of this study only covers 3 counties in Guizhou Province, so caution should be exercised when extrapolating the conclusions to other provinces; (2) Cross-sectional data cannot reveal the long-term changes in behavioral intention; (3) Moderating variables such as age, digital literacy, and health literacy are not included, and the model can be further improved; (4) This study selected patients in county-level hospitals as survey subjects, mainly considering that patients in hospital scenarios have more direct contact with medical services and higher questionnaire response rates. However, there are certain limitations: high-risk home groups such as home-bound elderly, bedridden chronic disease patients, and mobility-impaired people are not included. (5) Common method bias, though tested, cannot be fully eliminated. (6) Usage intention is self-reported and may not fully reflect actual behavior. (7) No objective platform usage data was available for validation. (8) Potential social desirability bias may affect self-reported attitudes toward digital health. (9) Measurement invariance was not tested for multi-group analysis.

Prospects: (1) Conduct multi-province and large-sample surveys (community home groups) in the future to improve the universality of conclusions; (2) Adopt longitudinal tracking research to explore the dynamic evolution of usage behavior; (3) Incorporate variables such as health literacy and digital divide to construct a more comprehensive influencing factor model; (4) Conduct comparative research on segmented services such as online drug purchase, Internet hospitals, and chronic disease management.

## Data Availability

The raw data supporting the conclusions of this article will be made available by the authors, without undue reservation.
